# Par‐aPKC‐dependent and ‐independent mechanisms cooperatively control cell polarity, Hippo signaling, and cell positioning in 16‐cell stage mouse embryos

**DOI:** 10.1111/dgd.12235

**Published:** 2015-10-09

**Authors:** Yoshikazu Hirate, Shino Hirahara, Ken‐ichi Inoue, Hiroshi Kiyonari, Hiroshi Niwa, Hiroshi Sasaki

**Affiliations:** ^1^ Department of Cell Fate Control Institute of Molecular Embryology and Genetics Kumamoto University 2‐2‐1 Honjo, Chuo‐ku Kumamoto 860‐0811 Japan; ^2^ Laboratory for Embryonic Induction RIKEN Center for Developmental Biology 2‐2‐3 Minatojima‐minamimachi, Chuo‐ku Kobe Hyogo 650‐0047 Japan; ^3^ Animal Resource Development Unit Division of Bio‐function Dynamics Imaging RIKEN Center for Life Science Technologies 2‐2‐3 Minatojima‐minamimachi, Chuo‐ku Kobe Hyogo 650‐0047 Japan; ^4^ Genetic Engineering Team Division of Bio‐function Dynamics Imaging RIKEN Center for Life Science Technologies 2‐2‐3 Minatojima‐minamimachi, Chuo‐ku Kobe Hyogo 650‐0047 Japan; ^5^ Laboratory for Pluripotent Cell Studies RIKEN Center for Developmental Biology 2‐2‐3 Minatojima‐minamimachi, Chuo‐ku Kobe Hyogo 650‐0047 Japan; ^6^ Department of Stem Cell Biology Institute of Molecular Embryology and Genetics Kumamoto University 2‐2‐1 Honjo, Chuo‐ku Kumamoto 860‐0811 Japan; ^7^ Laboratory for Embryogenesis Graduate School of Frontier BioSciences Osaka University 1‐3 Yamadaoka Suita Osaka 565‐0871 Japan; ^8^ Present address: Center for Experimental Animals Tokyo Medical and Dental University 1‐5‐45 Yushima Bunkyo‐ku Tokyo 113‐8510 Japan

**Keywords:** asymmetric cell division, cell polarity, Hippo signaling, Par‐aPKC, preimplantation embryo

## Abstract

In preimplantation mouse embryos, the Hippo signaling pathway plays a central role in regulating the fates of the trophectoderm (TE) and the inner cell mass (ICM). In early blastocysts with more than 32 cells, the Par‐aPKC system controls polarization of the outer cells along the apicobasal axis, and cell polarity suppresses Hippo signaling. Inactivation of Hippo signaling promotes nuclear accumulation of a coactivator protein, Yap, leading to induction of TE‐specific genes. However, whether similar mechanisms operate at earlier stages is not known. Here, we show that slightly different mechanisms operate in 16‐cell stage embryos. Similar to 32‐cell stage embryos, disruption of the Par‐aPKC system activated Hippo signaling and suppressed nuclear Yap and Cdx2 expression in the outer cells. However, unlike 32‐cell stage embryos, 16‐cell stage embryos with a disrupted Par‐aPKC system maintained apical localization of phosphorylated Ezrin/Radixin/Moesin (p‐ERM), and the effects on Yap and Cdx2 were weak. Furthermore, normal 16‐cell stage embryos often contained apolar cells in the outer position. In these cells, the Hippo pathway was strongly activated and Yap was excluded from the nuclei, thus resembling inner cells. Dissociated blastomeres of 8‐cell stage embryos form polar–apolar couplets, which exhibit different levels of nuclear Yap, and the polar cell engulfed the apolar cell. These results suggest that cell polarization at the 16‐cell stage is regulated by both Par‐aPKC‐dependent and ‐independent mechanisms. Asymmetric cell division is involved in cell polarity control, and cell polarity regulates cell positioning and most likely controls Hippo signaling.

## Graphical abstract

In 16‐cell stage mouse embryos, cell polarity regulated by the Par‐aPKC system inhibits asymmetric cell division, thereby regulating the number of inner cells. In contrast to 32‐cell stage embryos, effects of the Par‐aPKC system on Yap activity and Cdx2 expression is limited at the 16‐cell stage.

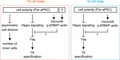

## Introduction

Before implantation in the uterus, mouse embryos undergo several rounds of cell division and form a cyst‐like structure called the blastocyst (Yamanaka *et al*. 2006; Sasaki 2010, 2015). The early blastocyst contains two types of cells, the trophectoderm (TE) and the inner cell mass (ICM). The TE is an outer epithelial structure required for implantation that later gives rise to placental tissues, whereas the ICM is a mass of pluripotent cells surrounded by the TE that later forms the embryo proper and some extraembryonic tissues.

Formation of the TE and ICM is the first cell fate specification in mouse development, and it has been the focus of scientific investigation for decades. Over the course of these studies, two models have emerged as central concepts in this field. One model is the “inside‐outside (positional) model” proposed by Tarkowski (Tarkowski & Wroblewska 1967), in which the position of the cells within the embryo determines the eventual fate of the cells; the outer layer of cells become the TE, and the inner cells become the ICM. The other model is the “polarity model” proposed by Johnson (Johnson *et al*. 1981), in which cell polarization at the 8‐cell stage and asymmetric inheritance of cell polarity during cell division regulates cell fate. At the 8‐cell stage, embryos undergo compaction, and cells establish apicobasal cell polarity (Johnson & McConnell 2004). In the next two rounds of cell division, the divisions are symmetric or asymmetric, contingent on the inheritance of the apical membrane (Graham & Deussen 1978; Johnson & Ziomek 1981a; Pedersen *et al*. 1986; Fleming 1987). In symmetric division, both daughter cells inherit the apical membrane, which is characterized by the presence of microvilli and maintenance of cell polarity. In asymmetric division, only one daughter cell inherits the apical membrane and maintains cell polarity, whereas the other loses the apical membrane and becomes apolar. Recently, a newer version of the polarity model was proposed, in which the presence of cell polarity or the apical domain regulates cell fate by controlling gene expression independent of cell division (Yamanaka *et al*. 2006).

Recent studies have revealed that Hippo signaling plays a central role in this cell‐fate specification process and have provided a molecular basis for the historical models (Nishioka *et al*. 2009; Cockburn *et al*. 2013; Hirate *et al*. 2013; Leung & Zernicka‐Goetz 2013; Hirate & Sasaki 2014; Wicklow *et al*. 2014). Activation of the Hippo signaling pathway depends on the position of the cells; Hippo signaling is active in the inner cells and inactive in the outer cells. In the outer cells, inactive Hippo signaling promotes nuclear accumulation of the coactivator proteins Yap1 and Taz (hereafter collectively designated as Yap), which activates the transcription factor Tead4 by forming a Tead4‐Yap complex. Active Tead4 induces the TE‐specific transcription factor Cdx2, and expression of Cdx2 promotes TE development (Niwa *et al*. 2005; Strumpf *et al*. 2005; Dietrich & Hiiragi 2007; Ralston & Rossant 2008). In contrast, in the inner cells, activation of the Hippo pathway inhibits nuclear accumulation of Yap, and Tead4 remains inactive. As Tead4 does not induce Cdx2, these cells adopt the alternative ICM fate. This cell position‐dependent Hippo signaling provides evidence for the inside‐outside model.

Studies at the 32‐cell stage have revealed that position‐dependent Hippo signaling is established through a combination of cell–cell adhesion and cell polarity. The inner cells are apolar; therefore, cell–cell adhesion activates the Hippo pathway. At adherens junctions (AJs), a scaffold protein angiomotin (Amot), which associates with the intracellular domain of E‐cadherin through catenins and the tumor suppressor Nf2/Merlin, strongly interacts with the protein kinase Lats1/2 and activates the Hippo pathway (Cockburn *et al*. 2013; Hirate *et al*. 2013; Hirate & Sasaki 2014). The outer cells have apicobasal polarity, and cell polarity regulated by the Par‐aPKC system (Suzuki & Ohno 2006) sequesters Amot from basolateral AJs to the apical domains (Hirate *et al*. 2013; Hirate & Sasaki 2014). Due to the absence of Amot in the E‐cadherin complex, cell–cell adhesion does not activate the Hippo pathway. Regulation of Hippo signaling by cell polarity indicates that the newer version of the polarity model can explain the molecular events that unfold at the 32‐cell stage.

Although the mechanisms that control position‐dependent Hippo signaling at the 32‐cell stage have been extensively studied, it is unclear if these mechanisms also operate at earlier stages. It is important to elucidate the mechanisms acting at the 16‐cell stage, because inner cells are first formed during the 8‐ to 16‐cell transition (Morris *et al*. 2010). Recently, a correlation was reported between cell polarity and Hippo signaling in 16‐cell stage embryos (Anani *et al*. 2014). However, the roles of cell polarity in Hippo signaling have not been directly examined.

In this study, we investigated the role of cell polarity at the 16‐cell stage. Disruption of the Par‐aPKC system had limited effects on cell polarity and regulation of the Hippo signal/Yap. We also found that 16‐cell stage embryos often contain apolar and Hippo‐active cells in the outer position. These results highlight the differences in the regulation of cell polarity and Hippo signaling between 16‐ and 32‐cell stage embryos. The relationship between these results and historical models of embryo development is also discussed.

## Materials and methods

### Mouse lines

Wild‐type embryos were obtained by crossing F1 (C57BL/6xDBA/2; BDF1) female mice with C57BL/6 or BDF1 male mice. Mice were housed in environmentally controlled rooms in the Laboratory Animal Housing Facility of the RIKEN Center for Developmental Biology (CDB) and the Center for Animal Resources and Development (CARD) of Kumamoto University. All experiments were carried out according to the regulations for animal and recombinant DNA experiments of RIKEN CDB and Kumamoto University and the laws and protocols outlined by the Japanese government. All studies were approved by the institutional committees for animal and recombinant DNA experiments at the RIKEN CDB and Kumamoto University.

### Embryo culture and embryo manipulation

One‐ or two‐cell stage embryos were recovered from oviducts following standard protocols (Hogan *et al*. 1994) and were cultured as previously described (Nishioka *et al*. 2008). Dissociation of 8‐cell stage embryos was performed as previously described (Suwinska *et al*. 2008).

### RNA injection

Poly(A)‐tailed RNA was injected into both blastomeres of 2‐cell stage embryos as previously described (Nishioka *et al*. 2009). For dominant‐negative experiments, RNA encoding dnPKC*λ* was injected at a concentration of 400 ng/μL.

### Gene knockdown by injection of shRNA‐encoding plasmids

Gene knockdown was achieved by pronuclear injection of verified shRNA plasmids (Sigma‐Aldrich, MO, USA), as described previously (Alarcon 2010; Hirate *et al*. 2013). Plasmids were purified by electrophoresis on a low melting point agarose gel, followed by agarose digestion with a Thermostable *β*‐agarase (Wako pure chemical industries, Osaka, Japan) (Hogan *et al*. 1994). The purified plasmid DNA solution (10 ng/μL) was injected into male pronuclei according to standard protocols (Hogan *et al*. 1994).

### Immunofluorescent staining

Immunofluorescent staining of preimplantation embryos was performed as previously described (Nishioka *et al*. 2009), with slight modifications. Primary antibodies against Yap (Abnova, H00010413‐M01), p‐Yap (Cell Signaling, 4911), p‐ERM (Cell Signaling, 3141), PKC*λ*/*ζ* (Santa Cruz, sc‐216), E‐cadherin (Takara, M108), Pard6b (Santa Cruz, sc‐67393), Scribble (Santa Cruz, sc‐28737), Cdx2 (BioGenex, MU392‐UC), and Amot (Hirate *et al*. 2013) were used. For secondary antibodies, goat anti‐mouse, anti‐rabbit, or anti‐rat IgG antibodies labeled with either Alexa Fluor 488, 555, or 647 (Invitrogen) were used. For nuclear staining, Hoechst33258 (Dojindo, Kumamoto, Japan) was used. Confocal images were obtained by using either a Zeiss LSM 510 or Nikon A1 microscope.

### Quantitative data analysis and statistics

Quantification of the signal intensities in confocal images was performed using ImageJ software. Statistical analyses were performed using Prism 6 software (Graphpad, CA, USA).

## Results

### Polarization correlates with the nuclear localization of Yap

**Figure 1:**
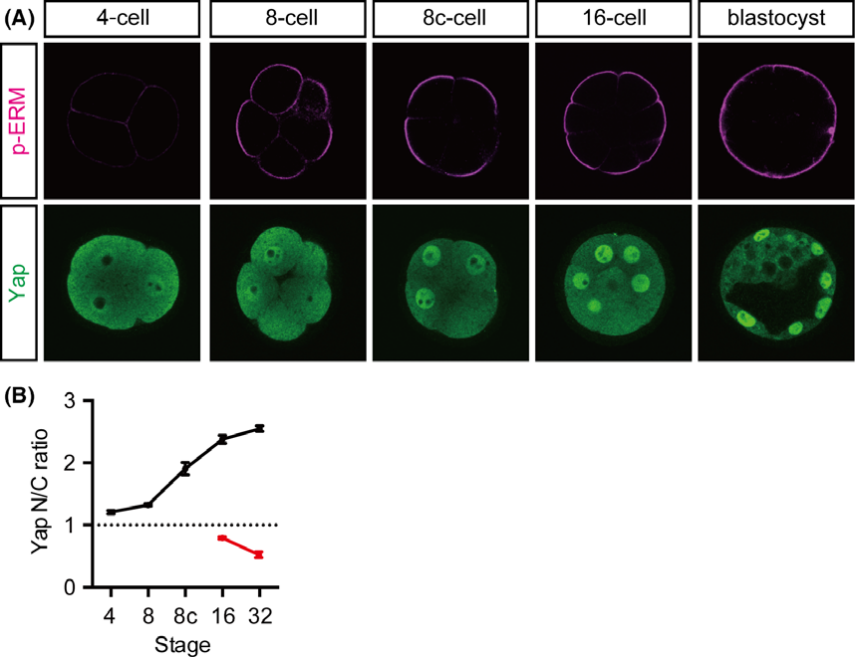
Correlation between cell polarity and nuclear Yap levels during preimplantation development. (A) Distribution of an apical marker, phosphorylated ERM (p‐ERM), and Yap between the 4‐cell and blastocyst stages. 8c‐cell, compacted 8‐cell stage. (B) Changes in the ratio of nuclear to cytoplasmic Yap (Yap N/C ratio) in the outer cells (black) and the inner cells (red). An N/C ratio of 1 indicates that the intensity of the Yap signal in the nucleus and cytoplasm is the same. When the N/C ratio is >1, Yap is predominantly nuclear. Graphs show the mean ± SEM (4‐cell, *n *= 20; 8‐cell, *n *= 8; 8c‐cell, *n *= 8; 16‐cell, *n *= 145 and 12 for outer and inner cells, respectively; 32‐cell, *n *= 174 and 83 for outer and inner cells, respectively).

To determine the role of cell polarity in the subcellular distribution of Yap at the 16‐cell stage, we first examined the correlation between cell polarity and the levels of nuclear Yap. Mouse embryos start to develop cell polarity at the 8‐cell stage after compaction (Johnson *et al*. 1986; Fleming & Johnson 1988). Polarization was visualized as the distribution of phosphorylated‐Ezrin/Radixin/Moesin (p‐ERM) proteins, which are localized to the apical microvilli (Fig. 1A) (Yonemura *et al*. 1999). The p‐ERM proteins were first detected throughout the plasma membrane in uncompacted 8‐cell stage embryos. After compaction, the strong signal was mostly restricted to the apical membrane. Nuclear Yap also became evident in the outer cells after compaction (Fig. 1A). To quantify the levels of Yap in the nuclei, we determined the nuclear to cytoplasmic (N/C) ratio of the Yap signals. Basal levels of nuclear Yap were observed from the 1‐cell stage. In the outer cells, the nuclear Yap signal began to increase after compaction and kept increasing over time, whereas in the inner cells, it decreased over time (Fig. 1B) as previously reported (Nishioka *et al*. 2009). The coincidence of the emergence of cell polarity and the increase in the nuclear Yap signal at the 16‐cell stage likely reflects their close relationship.

### Nuclear localization of Yap is partially perturbed by disruption of the Par‐aPKC system in 16‐cell stage embryos

**Figure 2:**
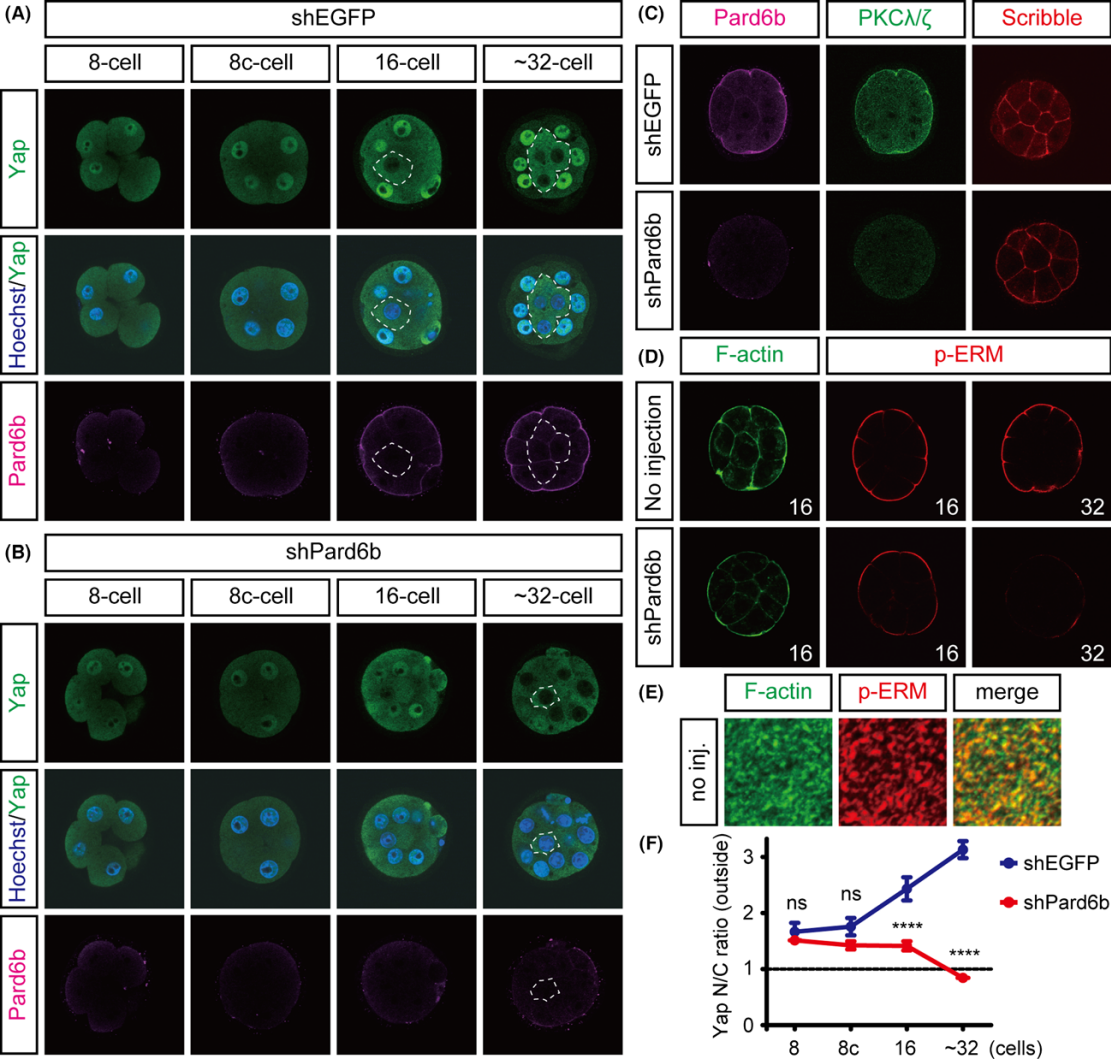
Effects of PAR‐aPKC system disruption on cell polarity and Yap localization. (A, B) Distribution of Yap and Pard6b proteins in embryos between the 8‐ and 32‐cell stages. (A) Distribution of Yap and Pard6b proteins in shEGFP‐injected embryos. (B) Distribution of Yap and Pard6b proteins in shPard6b‐injected embryos. Hoechst33258 (blue) was used to visualize nuclei. Dotted lines delineate the inner cells. (C) Distribution of Pard6b, PKC *λ*/*ζ*, and Scribble proteins in shEGFP and shPard6b‐injected embryos at the 16‐cell stage. (D) Distribution of F‐actin and p‐ERM in control (no injection) and shPard6b‐injected embryos at the 16‐ and 32‐cell stages. The number in each panel indicates the number of cells in the embryos. (E) Distribution of cortical F‐actin and p‐ERM in normal 16‐cell stage embryos. Stacked confocal images are shown. Note that the signals overlap in a dot‐like pattern. (F) Changes in the N/C ratio of Yap in the outer cells of shEGFP‐ and shPard6b‐injected embryos between the 8‐ and 32‐cell stages. Graphs show the mean ± SEM (*n *= 3 at each point except for shPard6b 16‐cell, where *n *= 4). ns, not significant; ****, *P *< 0.0001 vs. shEGFP‐injected embryos (two‐way ANOVA and Bonferroni's multiple comparisons test).

To directly examine the involvement of cell polarity in the regulation of Yap at the 16‐cell stage, we knocked down *Pard6b*, a component of the Par‐aPKC system. This was done by injecting a *Pard6b* shRNA expression plasmid. Following knockdown, we observed the development of the embryos. Consistent with previous observations, in control embryos, Pard6b gradually accumulated at the apical membrane during compaction and remained there in the outer cells up to the 32‐cell stage (Fig. 2A) (Vinot *et al*. 2005). In *Pard6b*‐knockdown (KD) embryos, apical Pard6b was clearly reduced by the 16‐cell stage (Fig. 2B). Loss of an apical regulator PKC*ζ*/*λ* (*n *= 7/7) and apical expansion of a basolateral regulator Scribble (*n *= 3/4) in *Pard6b*‐KD embryos confirmed disruption of the Par‐aPKC system at this stage (Fig. 2C). Reproducing previous results, the outer cells of *Pard6b*‐KD embryos showed clear exclusion of Yap from the nuclei at 32‐cell stage (Fig. 2B,F). In contrast, at the 16‐cell stage, *Pard6b*‐KD embryos did not clearly exclude Yap from the nuclei of the outer cells, even though the nuclear Yap signals were significantly weaker than those in control embryos (Fig. 2B,F). Therefore, the effects of disruption of the Par‐aPKC system on the regulation of Yap are weaker at the 16‐cell stage than at the 32‐cell stage.

### Cdx2 expression is moderately affected by disruption of the Par‐aPKC system at the 16‐cell stage

**Figure 3:**
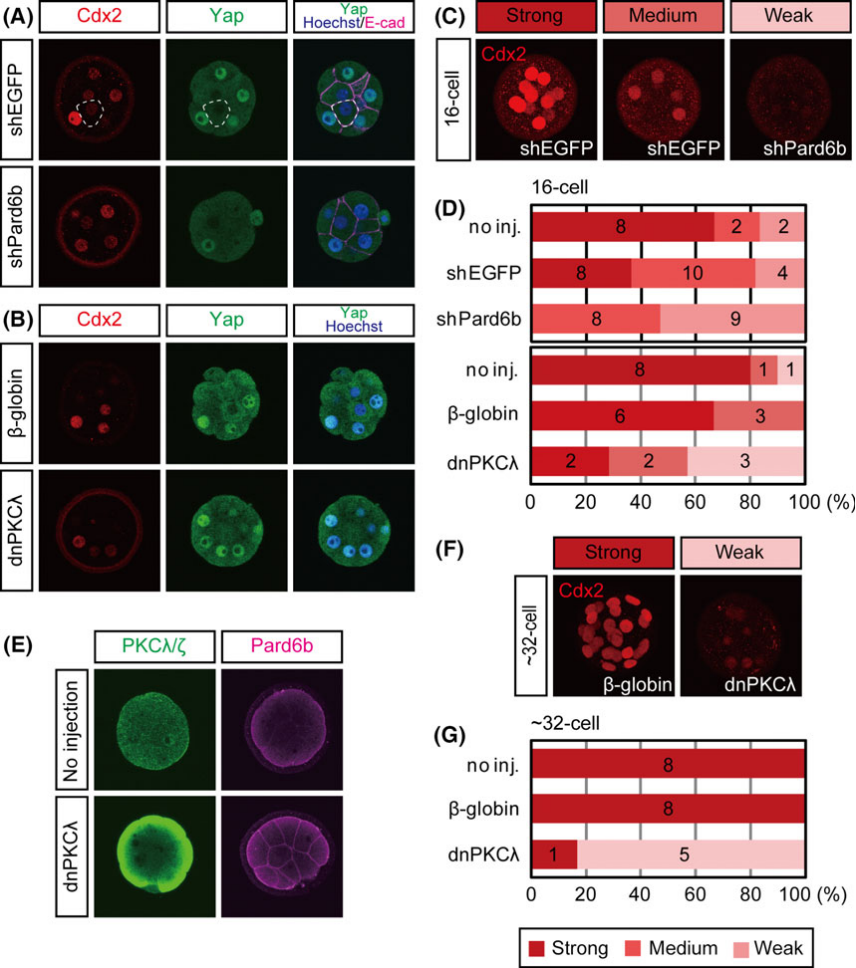
Effects of Par‐aPKC system disruption on Cdx2 expression. (A) Distribution of Cdx2 and Yap proteins in shEGFP‐ and shPard6b‐injected embryos. Nuclei were visualized with Hoechst33258. E‐cadherin was used to visualize the cell boundary. (B) Distribution of Cdx2 and Yap proteins in *β*‐globin‐ and dnPKC *λ*‐injected embryos. (C, D) Semi‐quantitative analyses of the effects of Par‐aPKC system disruption on the expression of Cdx2 at the 16‐cell stage. (C) Representative images of embryos showing strong, medium, and weak Cdx2 expression. Confocal images were projected to show total Cdx2 expression. (D) Graphs summarizing the expression of Cdx2 at the 16‐cell stage. The numbers in the graphs indicate the number of embryos in each category. (E) Distribution of PKC *λ*/*ζ* and Pard6b proteins in control and dnPKC *λ*‐injected embryos at the 16‐cell stage. (F, G) Semi‐quantitative analyses of the effects of Par‐aPKC system disruption on the expression of Cdx2 at the 32‐cell stage. (F) Representative images of embryos showing strong and weak Cdx2 expression. Confocal images were projected to show total Cdx2 expression. (G) Graphs summarizing the expression of Cdx2 at the 32‐cell stage. The numbers in the graphs indicate the number of embryos in each category.

At the 16‐cell stage, the effect of *Pard6b* KD on the subcellular localization of Yap was weak. Because Yap protein forms a complex with Tead4 in the nucleus and activates *Cdx2* enhancer, we hypothesized that the effect of *Pard6b* KD on the expression of Cdx2 would also be weak. Although *Pard6b*‐KD embryos showed reduced levels of Cdx2 at the 16‐cell stage (Fig. 3A,C,D), the reduction was less prominent than previously observed at the 32‐cell stage (Alarcon 2010; Hirate *et al*. 2013). These results supported our hypothesis.

To further confirm the weak effects of Par‐aPKC system disruption at the 16‐cell stage, we also used a second approach. We injected RNAs encoding the dominant‐negative form of PKC*λ* (dnPKC*λ*) into both blastomeres of two‐cell stage embryos. Similar to that observed in previous studies, overexpression of dnPKC*λ* resulted in clear exclusion of Yap from the nuclei and a strong reduction of Cdx2 at the 32‐cell stage (Fig. 3F,G) (Hirate *et al*. 2013). Immunostaining between the 8‐ and 16‐cell stages revealed strong, widespread expression of dnPKC*λ* protein (Fig. 3E; *n *= 6/6). Pard6b protein was distributed throughout the plasma membrane, indicating disruption of the Par‐aPKC system (Fig. 3E; *n *= 10/10). In 16‐cell stage embryos, the effects of dnPKC*λ* were similar to those of *Pard6b* KD; both the nuclear localization of Yap and the expression of Cdx2 were only moderately reduced (Figs 2F and 3B,D). These results suggest that the contribution of the Par‐aPKC system to the regulation of Yap and Cdx2 expression differs between the 16‐ and 32‐cell stages. Whereas the Par‐aPKC system determines the subcellular localization of Yap and strongly regulates the expression of Cdx2 at the 32‐cell stage, it has a limited role at the 16‐cell stage.

### Disruption of the Par‐aPKC system at the 16‐cell stage activates Hippo signaling in the outer cells

**Figure 4:**
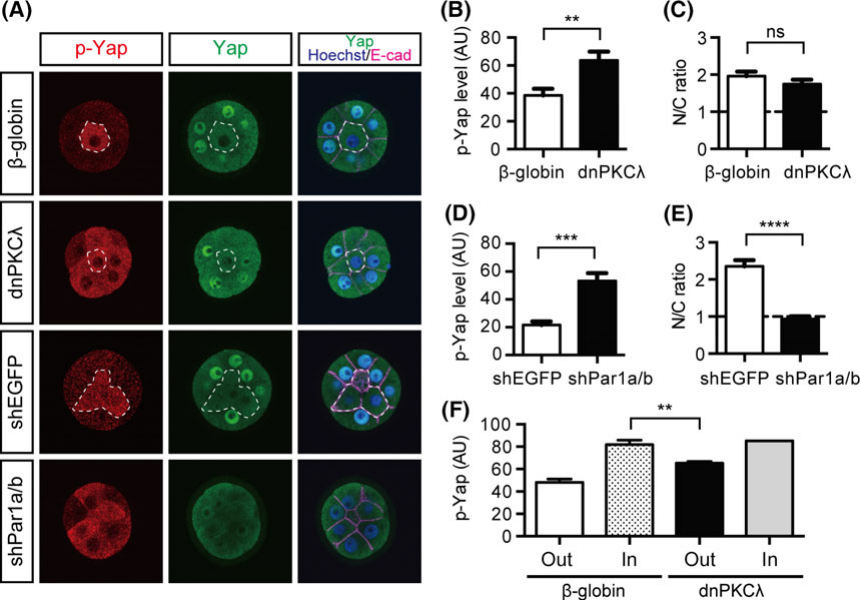
Effects of Par‐aPKC system disruption on Hippo signaling at the 16‐cell stage. (A) Distribution of phosphorylated Yap (p‐Yap) and Yap proteins in 16‐cell stage embryos. The nuclei were stained with Hoechst33258. Dotted lines delineate the inner cells. (B, C) Intensity levels of p‐Yap signals (B) and N/C ratio of Yap (C) in the outer cells of the *β*‐globin‐ and dnPKC *λ*‐injected embryos shown in (A). (D, E) Intensity levels of p‐Yap signals (D) and N/C ratio of Yap (E) in the outer cells of the shEGFP‐ and shPar1a/b‐injected embryos shown in (A). (F) Semi‐quantitative analysis of the p‐Yap signals from *β*‐globin‐ and dnPKC *λ*‐injected embryos (*n *= 5 each). Graphs show the mean ± SEM. ns, not significant; **, *P* < 0.01; ***, *P* < 0.001; ****, *P* < 0.0001 vs. the control (*β*‐globin or shEGFP‐injected) embryos (*t*‐test).

At the 16‐cell stage, disruption of the Par‐aPKC system resulted in limited exclusion of Yap from the nuclei and moderate Cdx2 expression in the outer cells. At the 32‐cell stage, disruption of the Par‐aPKC system activates Hippo signaling leading to clear exclusion of Yap from the nuclei in the outer cells (Hirate *et al*. 2013). Therefore, we hypothesized that activation of Hippo signaling was incomplete in polarity‐disrupted embryos at the 16‐cell stage. Upon activation of the Hippo pathway, protein kinase Lats1/2 phosphorylates five serine residues in Yap, including serine 112 (S112, which is equivalent to serine 127 in human Yap). To monitor Hippo signaling, we examined the distribution of S112‐phosphorylated Yap (p‐Yap). In control embryos, a strong p‐Yap signal was only observed in the inner cells (Fig. 4A; *n *= 20/20). In the dnPKC*λ*‐injected embryos, a strong p‐Yap signal was also observed in the outer cells, indicating activation of the Hippo pathway in these cells (Fig. 4A,B; *n *= 16/19). Double knockdown (DKD) of the basolateral regulators *Par1a* and *Par1b* by shRNA also resulted in increased p‐Yap levels in the outer cells (Fig. 4A,D; *n *= 20/20). Therefore, similar to that observed at the 32‐cell stage, disruption of the Par‐aPKC system ectopically activates Hippo signaling in the outer cells at the 16‐cell stage.

Despite the clear activation of Hippo signaling in the outer cells, neither dnPKC*λ*‐injected (*n *= 24/24) nor *Par1a/b*‐DKD (*n *= 20/29) embryos showed clear exclusion of Yap from the nuclei in the outer cells (Fig. 4A,C,E). We hypothesized that the increase in p‐Yap levels in the outer cells is less than that in the inner cells and is not sufficient to exclude Yap from the nuclei. Therefore, we compared the Yap phosphorylation levels in control and dnPKC*λ*‐injected embryos semi‐quantitatively by acquiring and analyzing p‐Yap signals under the same experimental conditions. Indeed, at the 16‐cell stage, the outer cells of dnPKC*λ*‐injected embryos (*n *= 5; 79 cells) showed lower p‐Yap levels than the inner cells of control embryos (*n *= 5; 8 cells; Fig. 4F). These results are consistent with the hypothesis that disruption of the Par‐aPKC system does not fully activate Hippo signaling in the outer cells to the extent required to exclude Yap from the nuclei at the 16‐cell stage.

### Disruption of the Par‐aPKC system does not completely disrupt the organization of the apical cortex at the 16‐cell stage

Because disruption of the Par‐aPKC system showed stage‐dependent differential effects on the regulation of Hippo signaling, Yap, and Cdx2, we investigated whether other stage‐dependent differences are also present in *Pard6b*‐KD embryos. We found that the effects of *Pard6b* KD on the apical p‐ERM signals differed depending on the developmental stage. As previously reported, at the 32‐cell stage, the apical p‐ERM signal was strongly reduced (Fig. 2D) (Hirate *et al*. 2013), whereas at the 16‐cell stage, the p‐ERM signal was not significantly altered (Fig. 2D). Phosphorylated or activated ERM proteins crosslink actin filaments and membrane proteins to promote the formation of microvilli in the apical domain of epithelial cells (Yonemura *et al*. 1999). Indeed, in the apical domain of 16‐cell stage embryos, p‐ERM signals were distributed as distinct dots or pillars perpendicular to the cortex, and these signals partially overlapped with the F‐actin signals (Fig. 2E). In *Pard6b*‐KD embryos, at the 16‐cell stage, as expected from the presence of strong apical p‐ERM signals, the F‐actin signals in the apical cortex were also largely unaffected, while the F‐actin signals in the basolateral cortex and the apical edge of cell–cell boundaries were clearly reduced (Fig. 2D). These results suggest that, at the 16‐cell stage, establishment and/or maintenance of the cortical apical pole, which consists of p‐ERM proteins, F‐actin, and microvilli, does not fully depend on the Par‐aPKC system. This implies that cell polarity at the 16‐cell stage is controlled by at least two mechanisms, the Par‐aPKC system and a yet unknown mechanism that controls the cortical apical pole independent of the Par‐aPKC system.

### The presence of outer apolar cells with intense Hippo signaling in 16‐cell stage embryos

**Figure 5:**
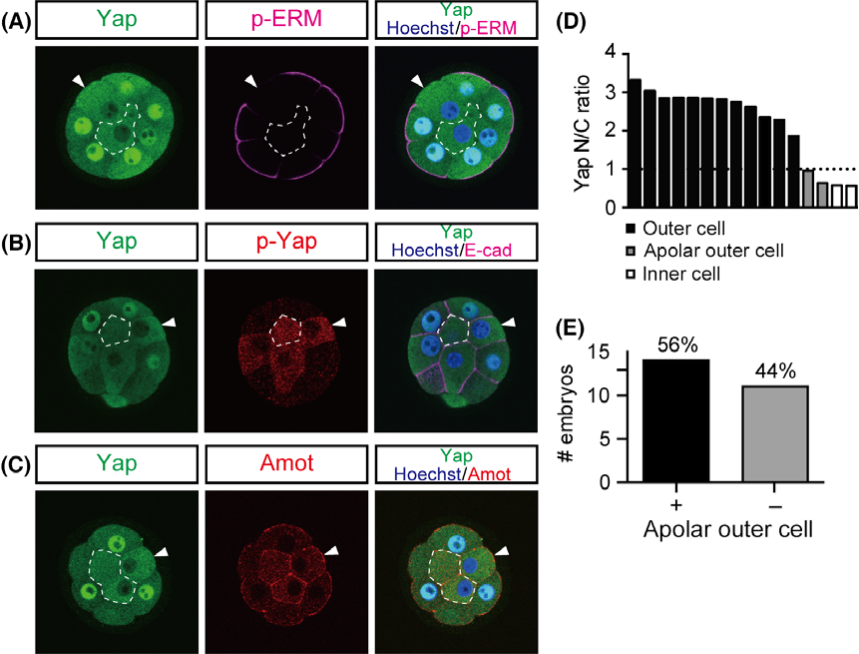
16‐cell stage embryos often contain apolar cells in the outer position. (A–C) Distribution of Yap, p‐ERM, p‐Yap, and Amot in normal embryos at the 16‐cell stage. Arrowheads indicate apolar cells at the outer position. Dotted lines delineate the inner cells. (D) Distribution of the Yap N/C ratio of each blastomere in a representative 16‐cell stage embryo containing apolar outer cells. (E) Number of embryos with (+) or without (−) apolar outer cells.

At the 32‐cell stage, subcellular localization of Yap always correlates with cell position in embryos, and manipulation of cell position alters Yap localization (Nishioka *et al*. 2009; Hirate *et al*. 2012). A correlation between Yap and cell position was also observed at the 16‐cell stage (Nishioka *et al*. 2009; Hirate *et al*. 2012). However, detailed examination of embryos at this stage revealed that 56% of embryos (*n *= 14/25) contain at least one outer cell that shows nuclear exclusion of Yap (Figs 5A,D,E and S1). These cells show strong p‐Yap signals, which are indicative of active Hippo signaling (Fig. 5B). Furthermore, these cells are apolar, as revealed by the absence of apical p‐ERM signals (Fig. 5A). Therefore, these Hippo‐active outer cells resemble inner cells. At the 32‐cell stage, the distribution of Amot is regulated by cell polarity. In the outer (polar) cells, Amot is restricted to the apical domain, whereas in the inner (apolar) cells, Amot is distributed throughout the plasma membrane (Hirate *et al*. 2013). In 16‐cell stage embryos, Hippo‐active outer cells also showed Amot throughout the plasma membrane (Fig. 5C), further confirming that these cells are apolar. Morphologically, the polar outer cells were flattened and their nuclei were located in the periphery of the embryo, whereas the apolar outer cells were columnar and their nuclei were more centrally located (Fig. 5A–C). Taken together, these results suggest that at the 16‐cell stage, some embryos have outer cells with characteristics of inner cells, such as the absence of cell polarity and active Hippo signaling.

### Asymmetric cell division regulates cell polarity, Yap, and cell positioning during the division of dissociated blastomeres of 8‐cell stage embryos

Studies at the 32‐cell stage revealed that cell position controls cell polarity and Hippo signaling. The presence of apolar and Hippo‐active cells in the outer position of 16‐cell stage embryos differentiates them from 32‐cell stage embryos. In 16‐cell stage embryos, cell position does not necessarily control cell polarity and Hippo signaling. Therefore, we hypothesized that the cell division processes during the 8‐ to 16‐cell transition are also involved in the regulation of cell polarization and Hippo signaling, independent of cell position.

**Figure 6:**
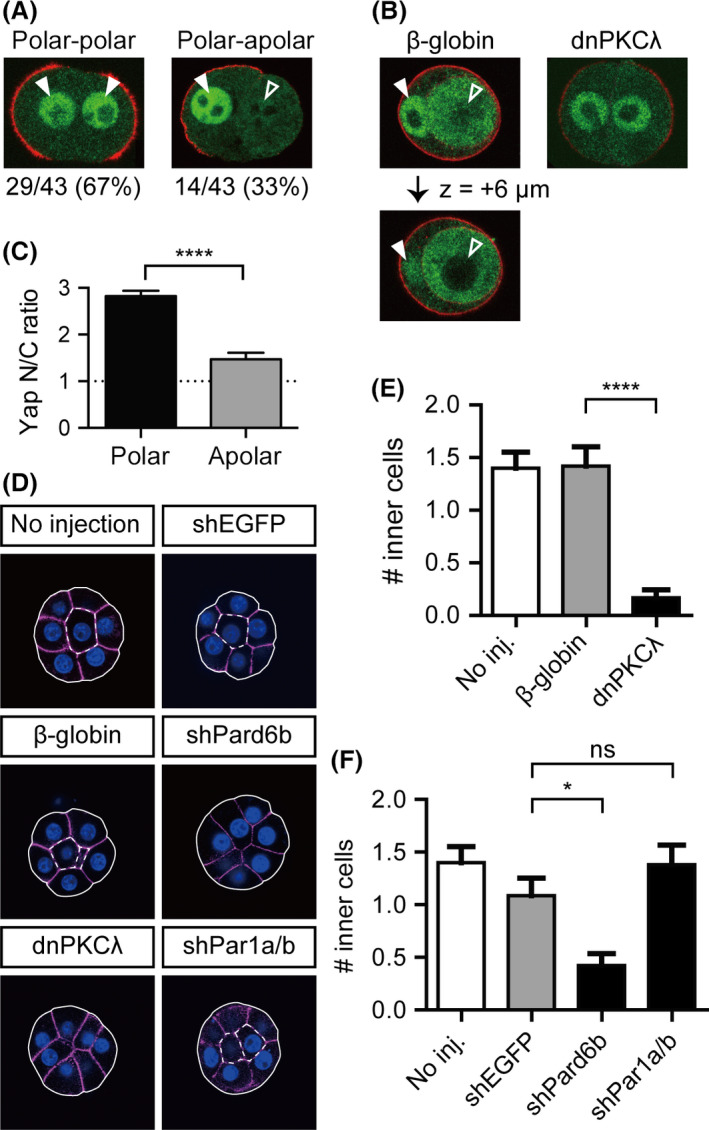
Relationship between cell division, cell polarity, Hippo signaling, and cell position. (A) Representative images of 2/16 couplets generated by cell division of 1/8 blastomeres after a 10‐h incubation. Yap and p‐ERM are shown in green and red, respectively. Filled and open arrowheads indicate the nuclei of polar and apolar cells, respectively. (B) Representative images of 2/16 couplets generated by cell division of 1/8 blastomeres after a 14‐h incubation. The polar cell of the polar‐apolar couplets from a control embryo engulfed the apolar cell, in which Yap was clearly excluded from the nucleus (left panel). The bottom panel shows a confocal slice, 6 μm above the z‐plane of the couplet shown in the top panel. No engulfment was observed in the 2/16 couplets from dnPKC *λ*‐injected embryos (right panel). Yap and p‐ERM are shown in green and red, respectively. Filled and open arrowheads indicate the nuclei of polar and apolar cells, respectively. (C) The Yap N/C ratio in polar (*n *= 72) and apolar (*n *= 14) cells in the couplets after a 10‐h incubation. (D) Presence of inner cells in control (no injection, *β*‐globin‐, shEGFP *‐*injected) and dnPKC *λ*‐, shPard6b*‐,* and shPar1a/b*‐*injected embryos. Solid and dotted lines delineate shapes of embryos and the inner cells, respectively. (E, F) Number of inner cells in embryos with disrupted Par‐aPKC systems at the 16‐cell stage. Inhibition of apical components by injection with dnPKC *λ* (*n *= 24) (E) and shPard6b (*n *= 26) (F) reduced the number of inner cells compared to that in *β*‐globin (*n *= 31) and shEGFP (*n *= 35) injected embryos, whereas inhibition of basolateral component by injection with shPar1a/b (*n *= 29) did not reduce the number of inner cells. Graphs show the mean ± SEM. ns, not significant; *, *P* < 0.05; ****, *P* < 0.0001 (C, *t*‐test; E and F, one‐way ANOVA and Tukey's multiple comparison test).

To test this hypothesis, we studied the process of the 8‐ to 16‐cell transition by culturing individual blastomeres of dissociated 8‐cell stage embryos (1/8 cells) until they divide and give rise to 2/16 couplets. As reported previously, cell division resulted in either couplets of two polar cells, in which p‐ERM signals were present in the outer cortex of both cells (symmetric division, 29/43; 67%), or couplets of polar and apolar cells, in which only one cell showed a p‐ERM signal in the outer membrane (asymmetric division, 14/43; 33%; Fig. 6A) (Johnson & McConnell 2004; Dietrich & Hiiragi 2007). Cell positions are equivalent in freshly formed couplets, and the generation of polar‐apolar couplets suggests that cell division controls cell polarization in dissociated blastomeres.

In polar‐polar couplets, Yap was distinctly nuclear in both cells (Fig. 6A). In polar‐apolar couplets, only the polar cells marked by apical p‐ERM signals showed strong nuclear Yap signals, and the apolar cells showed weaker nuclear Yap signals (Fig. 6A). Therefore, Hippo signaling correlates with cell polarity. While apolar cells in 16‐cell stage embryos showed clear exclusion of Yap from the nuclei, the apolar cells of the freshly formed polar‐apolar 2/16 couplets did not exclude Yap from the nuclei (Fig. 6A,C). This difference is likely caused by the limited cell–cell adhesion in couplets and consequent insufficient activation of Hippo signaling. Indeed, after longer incubation, the apolar cells of 2/16 couplets were engulfed by the polar cells and Yap was clearly excluded from the nuclei of the internalized apolar cells (*n *= 4/8 polar‐apolar couplets; Fig. 6B).

All the couplets generated from dnPKC*λ*‐injected embryos underwent symmetric cell division (*n *= 17/17), suggesting that the Par‐aPKC system is required for asymmetric cell division. In these couplets, both cells exhibited weak p‐ERM signals. This is different from that observed in embryos with disrupted Par‐aPKC systems, in which strong apical p‐ERM signals were maintained (Fig. 2D). Because cell–cell adhesion restricts the positioning of microvilli (Johnson & Ziomek 1981b), this difference may reflect the requirement of cell–cell adhesion for the establishment and/or maintenance of apical p‐ERM in the absence of the Par‐aPKC system.

The couplets generated from dnPKC*λ*‐injected embryos did not undergo engulfment (*n *= 0/17). Therefore, polar cells with active Par‐aPKC systems are required to internalize the apolar cells. In further support of this observation, disruption of the Par‐aPKC system in embryos after injection of dnPKC*λ* or shPard6b reduced the number of inner cells at the 16‐cell stage (Fig. 6D–F) (Dard *et al*. 2009). The Par‐aPKC system establishes apicobasal cell polarity through mutual inhibition of the apical regulator complex (Par3‐Par6‐aPKC) and the basolateral regulator Par‐1 (Suzuki & Ohno 2006). Because disruption of the apical regulator complex by targeting aPKC and Pard6b compromised cell positioning, we next asked whether disruption of the basolateral regulator has similar effects. Remarkably, when the basolateral regulators *Par‐1a/b* were knocked down, a decrease in the number of inner cells was not observed. This result suggests that the apical Par‐aPKC complex is required for engulfment and internalization of the apolar cells. In summary, at the 16‐cell stage, cell division, which is regulated by the Par‐aPKC system, is an important determinant of cell polarity. Cell polarity correlates with the subcellular localization of Yap, and the apical Par‐aPKC complex controls the positioning of cells within embryos.

## Discussion

### Cell polarity but not cell position regulates Hippo signaling in 16‐cell stage embryos

Previous studies on Hippo signaling have shown that at the 32‐cell stage, cell position determines whether cells acquire polarity, and the resulting cell polarity, in combination with cell–cell adhesion, establishes position‐dependent Hippo signaling (Nishioka *et al*. 2009) (see Introduction). Therefore, the evidence from studies of 32‐cell stage embryos (Hirate *et al*. 2013; Hirate & Sasaki 2014) supports the inside‐outside model (Tarkowski & Wroblewska 1967) and the newer version of the polarity model (Yamanaka *et al*. 2006). In this study, we examined whether similar mechanisms also operate at 16‐cell stage, and we found several differences.

The first important difference is that 16‐cell stage embryos often contain apolar, Hippo‐active cells, i.e., cells with characteristics of inner cells, in the outer position. Therefore, at least for these cells, cell position determines neither cell polarity nor Hippo signaling. In experiments examining 2/16 couplets, some 1/8 blastomeres formed polar‐apolar couplets, indicating that the process of cell division (asymmetric cell division) plays a role in the regulation of cell polarity. In both normal 16‐cell stage embryos and 2/16 couplets, polar and apolar cells always exhibited inactive and active Hippo signaling, respectively. These observations are consistent with the hypothesis that cell polarity regulates Hippo signaling. Indeed, partial disruption of cell polarity through inhibition of the Par‐aPKC system altered Hippo signaling in 16‐cell stage embryos. Therefore, cell division is an important regulator of cell polarity, and cell polarity likely controls Hippo signaling.

The nuclei of the apolar, Hippo‐active outer cells in 16‐cell stage embryos are located in the deep interior of these cells. Therefore, they are probably identical to the transient outer cells that have been observed in time‐lapse imaging of nuclei (McDole *et al*. 2011). Recently, gradual internalization of apolar outer cells at the 16‐cell stage was observed by live cell tracking (Anani *et al*. 2014). Furthermore, in polar‐apolar 2/16 couplets, the apolar cell was engulfed by the polar cell, and embryos with disrupted Par‐aPKC systems have fewer inner cells at the 16‐cell stage. Therefore, cell polarity regulated by the Par‐aPKC system also controls cell position in embryos. These results demonstrate that at the 16‐cell stage or during the 8‐ to 16‐cell transition, cell division is involved in the regulation of cell polarity, cell polarity likely controls Hippo signaling, and the Par‐aPKC system controls cell position. This implies that the original polarity model, in which asymmetric cell division generates differences in cell polarity, as well as the newer version of the polarity model, in which cell polarity controls gene expression, are supported by our findings at the 16‐cell stage. However, unlike at the 32‐cell stage, the inside‐outside model cannot explain our observations at the 16‐cell stage.

### The effects of disruption of the Par‐aPKC system in 16‐ and 32‐cell stage embryos are different

**Figure 7:**
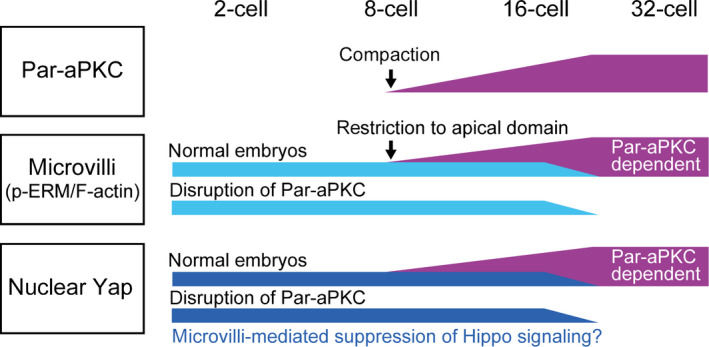
A model showing the relationship between the Par‐aPKC system, the cortical apical domain, represented by microvilli, and nuclear Yap. At the 16‐cell stage, both the Par‐aPKC system and cortical apical domain, represented by microvilli (p‐ERM/F‐actin), controls cell polarity and Yap localization. At the 32‐cell stage, the Par‐aPKC system plays a dominant role. See the Discussions for details.

The second important difference between the 16‐ and 32‐cell stages is the role of the Par‐aPKC system in the regulation of cell polarity and Hippo signaling. Disruption of the Par‐aPKC system in 32‐cell stage embryos completely disrupted cell polarity, activated Hippo signaling, and excluded Yap from the nuclei of the outer cells (Hirate *et al*. 2013). In contrast, at the 16‐cell stage, embryos with a disrupted Par‐aPKC system clearly maintained apical p‐ERM and F‐actin, the key regulators of apical microvilli, and Yap was not excluded from the nuclei of the outer cells. These results show two important differences between the 16‐ and 32‐cell stages (summarized in Fig. 7).

First, at the 16‐cell stage, p‐ERM and F‐actin, key components of microvilli, are not tightly regulated by the Par‐aPKC system. Consistent with this notion, microvilli are present at the 2‐cell stage, prior to initiation of the Par‐aPKC system at the 8‐cell stage (Calarco & Epstein 1973; Ducibella *et al*. 1977; Reeve & Ziomek 1981). At the early 8‐cell stage, cell–cell contacts restrict the position of microvilli. However, after 4–5 h, the position of the microvilli becomes fixed (Johnson & Ziomek 1981b), and they remain localized during mitotic division. It is likely that once fixed, microvilli, which consist of F‐actin, p‐ERM, and membrane proteins, constitute a stable cortical apical architecture that is independent of the Par‐aPKC system. Therefore, we propose that cell polarity in 16‐cell stage embryos is regulated by two independent mechanisms, the cortical apical domain, represented by microvilli/p‐ERM/F‐actin, and the Par‐aPKC system. After the 32‐cell stage, p‐ERM becomes dependent on the Par‐aPKC system.

Second, at the 16‐cell stage, the effects of disruption of the Par‐aPKC system on the regulation of Yap were not evident. Importantly, embryos with disrupted Par‐aPKC systems exhibited clear activation of Hippo signaling in the outer cells, suggesting that the mechanisms by which Par‐aPKC suppresses Hippo signaling in the outer cells commonly operate at both the 16‐ and 32‐cell stages. In support of this hypothesis, Amot, the junctional Hippo component that connects the Par‐aPKC system and Hippo signaling at the 32‐cell stage, shows a similar cell position‐dependent distribution at both the 16‐ and 32‐cell stages (Hirate *et al*. 2013). Then, the question that arises is why activation of the Hippo pathway does not promote exclusion of Yap from the nuclei. Since both the cortical apical pole, which is characterized by dense stable microvilli (Johnson & Mcconnell 2004), and nuclear Yap (Nishioka *et al*. 2009) are important in the regulation of cell fate, it is likely that the remaining apical microvilli contribute to the regulation of Yap. F‐actin is known to promote the nuclear localization of Yap by inhibiting Lats kinase (Sansores‐Garcia *et al*. 2011; Wada *et al*. 2011; Yu *et al*. 2012; Zhao *et al*. 2012). Because F‐actin bundles are the major components of microvilli, these F‐actins may contribute to the suppression of Hippo signaling and promote nuclear localization of Yap. Indeed, activation of Hippo signaling in the outer cells of Par‐aPKC‐disrupted 16‐cell stage embryos was weaker than that in the inner cells of normal 16‐cell stage embryos. One candidate molecule that connects microvillar F‐actin to Hippo signaling is Amot. While Amot activates Hippo signaling at adherens junctions, Amot activity is suppressed by its interaction with F‐actin (Hirate *et al*. 2013; Hirate & Sasaki 2014; Mana‐Capelli *et al*. 2014). In 32‐cell stage embryos, Amot localizes to microvilli and is required for suppression of Hippo signaling in the outer cells (Hirate & Sasaki 2014). Therefore, it is likely that F‐actin in microvilli restricts the distribution of Amot in the absence of the Par‐aPKC system, which reduces Hippo signaling.

Considering the small differences between the Hippo signaling level in the outer cells of the Par‐aPKC‐disrupted embryos and that in the inner cells of normal cells, it is also possible that the remaining apical microvilli, p‐ERM, and/or F‐actin promote nuclear localization of Yap independent of Hippo signaling through yet unknown mechanisms. In either case, the involvement of apical microvilli, p‐ERM, and/or F‐actin should be directly examined in the future by experimentally manipulating these molecules.

### Multiple mechanisms cooperatively establish the TE and ICM lineages

Previous studies have established that the Hippo signaling pathway plays a central role in the regulation of TE and ICM fates (Nishioka *et al*. 2009; Cockburn *et al*. 2013; Hirate *et al*. 2013; Leung & Zernicka‐Goetz 2013; Hirate & Sasaki 2014; Wicklow *et al*. 2014). While our previous studies focused on the mechanisms that regulate the signaling pathway at the 32‐cell stage, our current study revealed the mechanisms that partially regulate Hippo signaling and Yap at the 16‐cell stage. Combining these findings with other known mechanisms, we propose a model for the role and regulatory function of the Hippo signaling pathway in the development of TE and ICM.

At the 8‐cell stage, embryos undergo compaction, and each blastomere acquires apicobasal cell polarity. Cell polarity is regulated by two independent mechanisms, the Par‐aPKC system and the cortical apical domain, which is represented by microvilli (p‐ERM/F‐actin). During the 8‐ to 16‐cell transition, some blastomeres undergo asymmetric cell division, which is regulated by the stable cortical apical domain and the Par‐aPKC system. The cells that inherit the apical domain become polar cells, in which Hippo signaling is suppressed and nuclear localization of Yap is promoted. The cells that do not inherit the apical domain become apolar cells, in which Hippo signaling is activated and Yap is excluded from the nuclei. Nuclear Yap increases the activity of Tead4, which initiates TE fate. Recently, Notch signaling has been reported to be involved in promoting TE fate. Tead4‐Yap and the Notch transcription factor RBPJ cooperatively activate the *Cdx2* enhancer (Rayon *et al*. 2014). Activation of Notch signaling is initially stochastic at the morula stage, which likely contributes to the stochastic expression of Cdx2 at this stage (Dietrich & Hiiragi 2007). Asymmetric cell division also includes asymmetric inheritance of *Cdx2* mRNA to the polar cells (Skamagki *et al*. 2013). The presence of early bias among blastomeres at the 4‐cell stage also contributes to the generation of different lineages (Piotrowska‐Nitsche *et al*. 2005). However, the relationship between early bias and cell division during the 8‐ to 16‐cell transition has yet to be elucidated. Notch signaling becomes gradually restricted to the outer cells by the blastocyst stage (Rayon *et al*. 2014). After the 32‐cell stage, continuous, position‐dependent Hippo signaling likely functions as a safeguard mechanism for the establishment of two distinct cell fates. Hippo signaling maintains and enhances the position‐dependent differences between TE and ICM cells until these fates are fixed at the late blastocyst stage.

## Supporting information


**Fig. S1.** Distribution of the Yap N/C ratio in each blastomere in 16‐cell stage embryos.

## References

[dgd12235-bib-0001] Alarcon, V. B. 2010. Cell polarity regulator PARD6B is essential for trophectoderm formation in the preimplantation mouse embryo. Biol. Reprod. 83, 347–358. 20505164 10.1095/biolreprod.110.084400 PMC2924801

[dgd12235-bib-0002] Anani, S. , Bhat, S. , Honma‐Yamanaka, N. , Krawchuk, D. & Yamanaka, Y. 2014. Initiation of Hippo signaling is linked to polarity rather than to cell position in the pre‐implantation mouse embryo. Development 141, 2813–2824. 24948601 10.1242/dev.107276

[dgd12235-bib-0003] Calarco, P. G. & Epstein, C. J. 1973. Cell surface changes during preimplantation development in the mouse. Dev. Biol. 32, 208–213. 4789692 10.1016/0012-1606(73)90233-9

[dgd12235-bib-0004] Cockburn, K. , Biechele, S. , Garner, J. & Rossant, J. 2013. The Hippo pathway member Nf2 is required for inner cell mass specification. Curr. Biol. 23, 1195–1201. 23791728 10.1016/j.cub.2013.05.044

[dgd12235-bib-0005] Dard, N. , Le, T. , Maro, B. & Louvet‐Vallee, S. 2009. Inactivation of aPKClambda reveals a context dependent allocation of cell lineages in preimplantation mouse embryos. PLoS ONE 4, e7117. 19768116 10.1371/journal.pone.0007117 PMC2741596

[dgd12235-bib-0006] Dietrich, J. E. & Hiiragi, T. 2007. Stochastic patterning in the mouse pre‐implantation embryo. Development 134, 4219–4231. 17978007 10.1242/dev.003798

[dgd12235-bib-0007] Ducibella, T. , Ukena, T. , Karnovsky, M. & Anderson, E. 1977. Changes in cell surface and cortical cytoplasmic organization during early embryogenesis in the preimplantation mouse embryo. J. Cell Biol. 74, 153–167. 873999 10.1083/jcb.74.1.153 PMC2109885

[dgd12235-bib-0008] Fleming, T. P. 1987. A quantitative analysis of cell allocation to trophectoderm and inner cell mass in the mouse blastocyst. Dev. Biol. 119, 520–531. 3803716 10.1016/0012-1606(87)90055-8

[dgd12235-bib-0009] Fleming, T. P. & Johnson, M. H. 1988. From egg to epithelium. Annu. Rev. Cell Biol. 4, 459–485. 3058163 10.1146/annurev.cb.04.110188.002331

[dgd12235-bib-0010] Graham, C. F. & Deussen, Z. A. 1978. Features of cell lineage in preimplantation mouse development. J. Embryol. Exp. Morphol. 48, 53–72. 581769

[dgd12235-bib-0011] Hirate, Y. & Sasaki, H. 2014. The role of angiomotin phosphorylation in the Hippo pathway during preimplantation mouse development. Tissue Barriers. 2, e28127. 24843842 10.4161/tisb.28127 PMC4022607

[dgd12235-bib-0012] Hirate, Y. , Cockburn, K. , Rossant, J. & Sasaki, H. 2012. Tead4 is constitutively nuclear, while nuclear vs. cytoplasmic Yap distribution is regulated in preimplantation mouse embryos. Proc. Natl Acad. Sci. USA 109, E3389–E3390; author reply E3391‐3382. 23169672 10.1073/pnas.1211810109 PMC3528498

[dgd12235-bib-0013] Hirate, Y. , Hirahara, S. , Inoue, K. , *et al*. 2013. Polarity‐dependent distribution of angiomotin localizes Hippo signaling in preimplantation embryos. Curr. Biol. 23, 1181–1194. 23791731 10.1016/j.cub.2013.05.014 PMC3742369

[dgd12235-bib-0014] Hogan, B. , Beddington, R. , Constantini, F. & Lacy, E. 1994. Manipulating the Mouse Embryo: Laboratory Manual, 2nd edn. Cold Spring Harbor Laboratory Press, New York.

[dgd12235-bib-0015] Johnson, M. H. & McConnell, J. M. 2004. Lineage allocation and cell polarity during mouse embryogenesis. Semin. Cell Dev. Biol. 15, 583–597. 15271304 10.1016/j.semcdb.2004.04.002

[dgd12235-bib-0016] Johnson, M. H. & Ziomek, C. A. 1981a. The foundation of two distinct cell lineages within the mouse morula. Cell 24, 71–80. 7237545 10.1016/0092-8674(81)90502-x

[dgd12235-bib-0017] Johnson, M. H. & Ziomek, C. A. 1981b. Induction of polarity in mouse 8‐cell blastomeres: specificity, geometry, and stability. J. Cell Biol. 91, 303–308. 7298724 10.1083/jcb.91.1.303 PMC2111944

[dgd12235-bib-0018] Johnson, M. H. , Pratt, H. P. M. & Handyside, A. H. 1981. The Generation and Recognition of Positional Information in the Preimplantation Mouse Embryo. In: Cellular and Molecular Aspects of Implantation (eds S. R. Glasser & D. W. Bullock ), pp. 55–74. Plenum Press, New York.

[dgd12235-bib-0019] Johnson, M. H. , Maro, B. & Takeichi, M. 1986. The role of cell adhesion in the synchronization and orientation of polarization in 8‐cell mouse blastomeres. J. Embryol. Exp. Morphol. 93, 239–255. 3090189

[dgd12235-bib-0020] Leung, C. Y. & Zernicka‐Goetz, M. 2013. Angiomotin prevents pluripotent lineage differentiation in mouse embryos via Hippo pathway‐dependent and ‐independent mechanisms. Nat. Commun. 4, 2251. 23903990 10.1038/ncomms3251 PMC3741640

[dgd12235-bib-0021] Mana‐Capelli, S. , Paramasivam, M. , Dutta, S. & McCollum, D. 2014. Angiomotins link F‐actin architecture to Hippo pathway signaling. Mol. Biol. Cell 25, 1676–1685. 24648494 10.1091/mbc.E13-11-0701 PMC4019498

[dgd12235-bib-0022] McDole, K. , Xiong, Y. , Iglesias, P. A. & Zheng, Y. 2011. Lineage mapping the pre‐implantation mouse embryo by two‐photon microscopy, new insights into the segregation of cell fates. Dev. Biol. 355, 239–249. 21539832 10.1016/j.ydbio.2011.04.024 PMC3119919

[dgd12235-bib-0023] Morris, S. A. , Teo, R. T. , Li, H. , Robson, P. , Glover, D. M. & Zernicka‐Goetz, M. 2010. Origin and formation of the first two distinct cell types of the inner cell mass in the mouse embryo. Proc. Natl Acad. Sci. USA 107, 6364–6369. 20308546 10.1073/pnas.0915063107 PMC2852013

[dgd12235-bib-0024] Nishioka, N. , Yamamoto, S. , Kiyonari, H. , *et al*. 2008. Tead4 is required for specification of trophectoderm in pre‐implantation mouse embryos. Mech. Dev. 125, 270–283. 18083014 10.1016/j.mod.2007.11.002

[dgd12235-bib-0025] Nishioka, N. , Inoue, K. , Adachi, K. , *et al*. 2009. The Hippo signaling pathway components Lats and Yap pattern Tead4 activity to distinguish mouse trophectoderm from inner cell mass. Dev. Cell 16, 398–410. 19289085 10.1016/j.devcel.2009.02.003

[dgd12235-bib-0026] Niwa, H. , Toyooka, Y. , Shimosato, D. , *et al*. 2005. Interaction between Oct3/4 and Cdx2 determines trophectoderm differentiation. Cell 123, 917–929. 16325584 10.1016/j.cell.2005.08.040

[dgd12235-bib-0027] Pedersen, R. A. , Wu, K. & Balakier, H. 1986. Origin of the inner cell mass in mouse embryos: cell lineage analysis by microinjection. Dev. Biol. 117, 581–595. 2428686 10.1016/0012-1606(86)90327-1

[dgd12235-bib-0028] Piotrowska‐Nitsche, K. , Perea‐Gomez, A. , Haraguchi, S. & Zernicka‐Goetz, M. 2005. Four‐cell stage mouse blastomeres have different developmental properties. Development 132, 479–490. 15634695 10.1242/dev.01602

[dgd12235-bib-0029] Ralston, A. & Rossant, J. 2008. Cdx2 acts downstream of cell polarization to cell‐autonomously promote trophectoderm fate in the early mouse embryo. Dev. Biol. 313, 614–629. 18067887 10.1016/j.ydbio.2007.10.054

[dgd12235-bib-0030] Rayon, T. , Menchero, S. , Nieto, A. , *et al*. 2014. Notch and Hippo converge on Cdx2 to specify the trophectoderm lineage in the mouse blastocyst. Dev. Cell 30, 410–422. 25127056 10.1016/j.devcel.2014.06.019 PMC4146744

[dgd12235-bib-0031] Reeve, W. J. & Ziomek, C. A. 1981. Distribution of microvilli on dissociated blastomeres from mouse embryos: evidence for surface polarization at compaction. J. Embryol. Exp. Morphol. 62, 339–350. 7276817

[dgd12235-bib-0032] Sansores‐Garcia, L. , Bossuyt, W. , Wada, K. , *et al*. 2011. Modulating F‐actin organization induces organ growth by affecting the Hippo pathway. EMBO J. 30, 2325–2335. 21556047 10.1038/emboj.2011.157 PMC3116287

[dgd12235-bib-0033] Sasaki, H. 2010. Mechanisms of trophectoderm fate specification in preimplantation mouse development. Dev. Growth Differ. 52, 263–273. 20100249 10.1111/j.1440-169X.2009.01158.x

[dgd12235-bib-0034] Sasaki, H. 2015. Position‐ and polarity‐dependent Hippo signaling regulates cell fates in preimplantation mouse embryos. Semin. Cell Dev. Biol. in press, doi:10.1016/j.semcdb.2015.05.003. 25986053

[dgd12235-bib-0035] Skamagki, M. , Wicher, K. B. , Jedrusik, A. , Ganguly, S. & Zernicka‐Goetz, M. 2013. Asymmetric localization of Cdx2 mRNA during the first cell‐fate decision in early mouse development. Cell Rep. 3, 442–457. 23375373 10.1016/j.celrep.2013.01.006 PMC3607255

[dgd12235-bib-0036] Strumpf, D. , Mao, C. A. , Yamanaka, Y. , *et al*. 2005. Cdx2 is required for correct cell fate specification and differentiation of trophectoderm in the mouse blastocyst. Development 132, 2093–2102. 15788452 10.1242/dev.01801

[dgd12235-bib-0037] Suwinska, A. , Czolowska, R. , Ozdzenski, W. & Tarkowski, A. K. 2008. Blastomeres of the mouse embryo lose totipotency after the fifth cleavage division: expression of Cdx2 and Oct4 and developmental potential of inner and outer blastomeres of 16‐ and 32‐cell embryos. Dev. Biol. 322, 133–144. 18692038 10.1016/j.ydbio.2008.07.019

[dgd12235-bib-0038] Suzuki, A. & Ohno, S. 2006. The PAR‐aPKC system: lessons in polarity. J. Cell Sci. 119, 979–987. 16525119 10.1242/jcs.02898

[dgd12235-bib-0039] Tarkowski, A. K. & Wroblewska, J. 1967. Development of blastomeres of mouse eggs isolated at the 4‐ and 8‐cell stage. J. Embryol. Exp. Morphol. 18, 155–180. 6048976

[dgd12235-bib-0040] Vinot, S. , Le, T. , Ohno, S. , Pawson, T. , Maro, B. & Louvet‐Vallee, S. 2005. Asymmetric distribution of PAR proteins in the mouse embryo begins at the 8‐cell stage during compaction. Dev. Biol. 282, 307–319. 15950600 10.1016/j.ydbio.2005.03.001

[dgd12235-bib-0041] Wada, K. , Itoga, K. , Okano, T. , Yonemura, S. & Sasaki, H. 2011. Hippo pathway regulation by cell morphology and stress fibers. Development 138, 3907–3914. 21831922 10.1242/dev.070987

[dgd12235-bib-0042] Wicklow, E. , Blij, S. , Frum, T. , *et al*. 2014. HIPPO pathway members restrict SOX2 to the inner cell mass where it promotes ICM fates in the mouse blastocyst. PLoS Genet. 10, e1004618. 25340657 10.1371/journal.pgen.1004618 PMC4207610

[dgd12235-bib-0043] Yamanaka, Y. , Ralston, A. , Stephenson, R. O. & Rossant, J. 2006. Cell and molecular regulation of the mouse blastocyst. Dev. Dyn. 235, 2301–2314. 16773657 10.1002/dvdy.20844

[dgd12235-bib-0044] Yonemura, S. , Tsukita, S. & Tsukita, S. 1999. Direct involvement of ezrin/radixin/moesin (ERM)‐binding membrane proteins in the organization of microvilli in collaboration with activated ERM proteins. J. Cell Biol. 145, 1497–1509. 10385528 10.1083/jcb.145.7.1497 PMC2133160

[dgd12235-bib-0045] Yu, F. X. , Zhao, B. , Panupinthu, N. , *et al*. 2012. Regulation of the Hippo‐YAP pathway by G‐protein‐coupled receptor signaling. Cell 150, 780–791. 22863277 10.1016/j.cell.2012.06.037 PMC3433174

[dgd12235-bib-0046] Zhao, B. , Li, L. , Wang, L. , Wang, C. Y. , Yu, J. & Guan, K. L. 2012. Cell detachment activates the Hippo pathway via cytoskeleton reorganization to induce anoikis. Genes Dev. 26, 54–68. 22215811 10.1101/gad.173435.111 PMC3258966

