# Supplementary figures and images for: Par‐aPKC‐dependent and ‐independent mechanisms cooperatively control cell polarity, Hippo signaling, and cell positioning in 16‐cell stage mouse embryos
Source: Dev Growth Differ. 2015 Oct 9;57(8):544–56. doi: 10.1111/dgd.12235 (PMC11520972; doi:10.1111/dgd.12235)

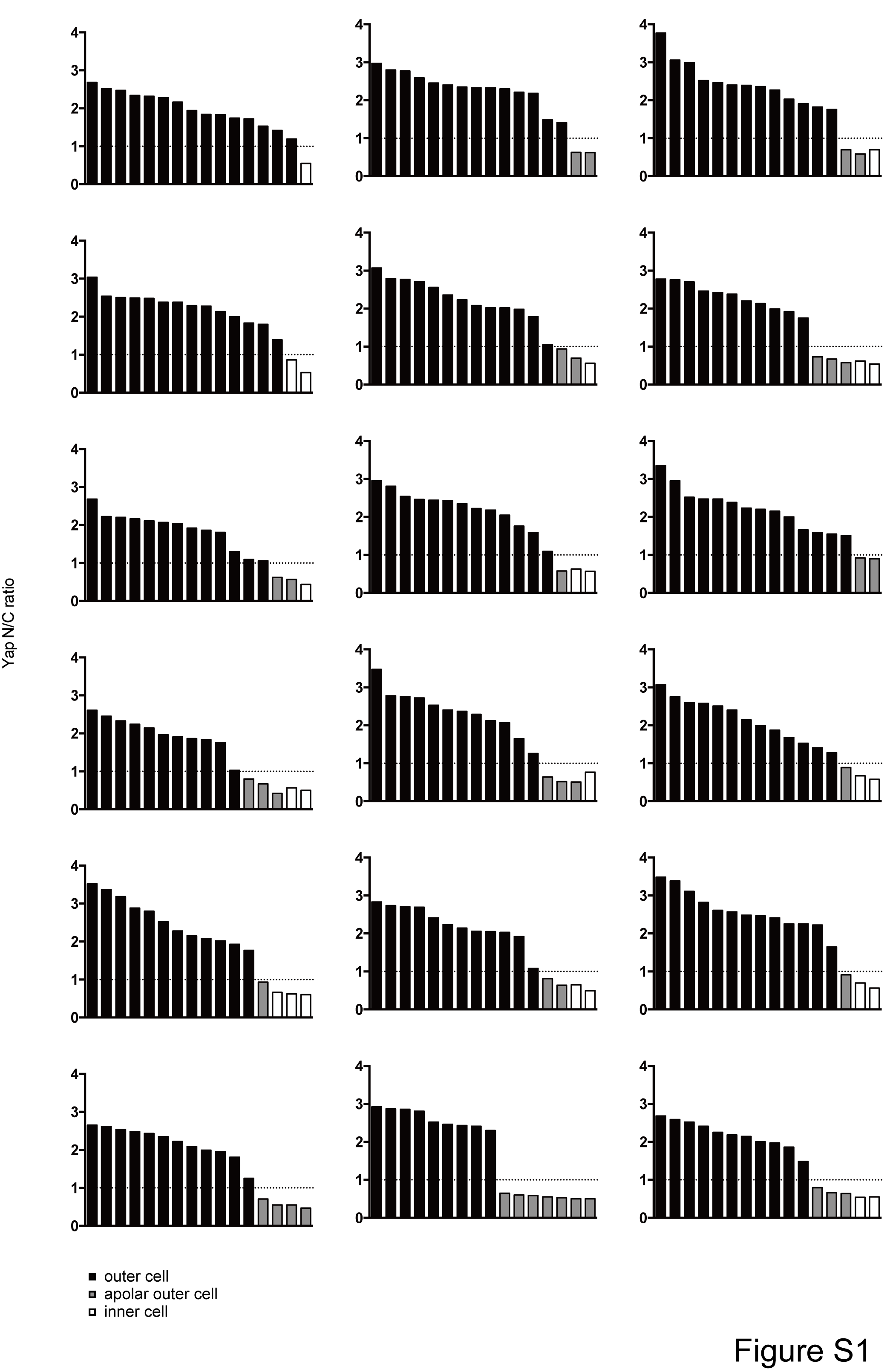

Supplement: Supplementary file 1 — Fig. S1. Distribution of the Yap N/C ratio in each blastomere in 16‐cell stage embryos. [file DGD-57-544-s001.tif]
